# Association between changes in brain microstructure and cognition in older subjects at increased risk for vascular disease

**DOI:** 10.1186/s12883-015-0396-z

**Published:** 2015-08-07

**Authors:** Michiel Sala, Albert de Roos, Gerard J. Blauw, Huub A. M. Middelkoop, J. Wouter Jukema, Simon P. Mooijaart, Mark A. van Buchem, Anton J. M. de Craen, Jeroen van der Grond

**Affiliations:** Department of Radiology, C3-Q, Leiden University Medical Center, Albinusdreef 2, 2333, ZA Leiden, The Netherlands; Gerontology and Geriatrics, Leiden, The Netherlands; Clinical Neuropsychology, Leiden, The Netherlands; Cardiology, Leiden University Medical Center, Leiden, The Netherlands; Consortium for Healthy Ageing, Leiden, The Netherlands

## Abstract

**Background:**

The purpose of this study is to investigate whether changes in brain microstructure, detected by magnetization transfer imaging, are associated with cognition in older subjects at increased risk for vascular disease.

**Methods:**

One hundred ninety three nondemented subjects (105 men, mean age 77 ± 3 years) from the Prospective Study of Pravastatin in the Elderly at Risk were included. To assess cross-sectional associations between magnetization transfer ratio (MTR) peak height and cognitive test scores, general linear model multivariate analysis was performed. Models were adjusted for age, sex, education level, vascular risk factors, individual white matter lesion volume, and brain atrophy. A repeated measures general linear model was used to investigate whether MTR peak height relates to cognitive test performance at baseline and 3.3-year follow-up.

**Results:**

Cross-sectionally, MTR peak height was associated with performance on the STROOP test (unstandardized β = −0.27, *p* = 0.045), delayed Picture Word Learning (PWL) test (β = 0.48, *p* = 0.007), and the Letter Digit Coding test (β = 1.1, *p* = 0.006). Repeated measures general linear model analysis showed that individuals with low MTR peak height at baseline performed worse on the STROOP test compared to subjects with intermediate MTR peak height (mean time to complete the test at baseline and follow-up, lower versus middle tertile of MTR peak height: 61.6 versus 52.7 s, *p* = 0.019) or compared to subjects with high MTR peak height (*p* = 0.046). Similarly, low MTR peak height was associated with worse performance on the immediate (lower versus middle tertile, *p* = 0.023; lower versus higher tertile, *p* = 0.032) and delayed PWL test (lower versus middle, *p* = 0.004; lower versus higher, *p* = 0.012) at baseline and follow-up testing.

**Conclusions:**

MTR peak height is associated with cognitive function in older subjects at increased risk for vascular disease.

**Electronic supplementary material:**

The online version of this article (doi:10.1186/s12883-015-0396-z) contains supplementary material, which is available to authorized users.

## Background

Age-related cognitive decline is of increasing concern among the elderly, still, the underlying mechanisms are incompletely understood [1]. Magnetic resonance imaging (MRI) enables in vivo characterization of visible cerebral changes occurring with both advancing age and cognitive decline, such as brain atrophy, the presence of small vessel disease, and basal ganglia iron depositions [2–4].

In addition to these overt MRI visible abnormalities, ‘hidden’ changes in brain tissue integrity can be assessed by magnetization transfer imaging [5]. It has been shown that lower magnetization transfer ratio (MTR) relates to decreasing myelin content and axonal count in both grey and white matter [6, 7]. There is a significant MTR decrease with advancing age [8, 9]. Currently, MTR metrics have been used extensively to evaluate brain microstructure in different neurodegenerative diseases [10–17].

Recently it has been shown that low MTR values correlate cross-sectionally with cognitive impairment in nondemented older subjects [18, 19]. However, no longitudinal studies have been performed investigating the association between MTR and cognitive changes over time.

Therefore at present it is unclear whether MTR has a role in predicting cognitive decline. Nevertheless, it has been put forward that MTR may be a useful outcome marker that is required to monitor disease progression and assess clinical intervention studies in demented and nondemented older subjects [18].

The purpose of this study is to investigate whether baseline MTR, a marker of brain microstructure, relates to cognition at baseline and 3.3-year follow-up in an older population at increased risk for vascular disease.

## Methods

The Prospective Study of Pravastatin in the Elderly at Risk (PROSPER study) has ethics review board approval of all locations. The individual ethics committees are listed in an additional file 1. Written informed consent was obtained from all participants. In addition, our local institutional ethics review board (the Medical Ethics Committee of the Leiden University Medical Center) approved the research protocol for the prospective MRI study and subsequent retrospective analyses. Participants of the current MRI study also agreed with retrospective analysis of their MRI data for research purposes.

### Study subjects

Subjects were included from the PROSPER Study which has been described in more detail elsewhere [20]. In short, subjects were enrolled based on the following inclusion criteria: (1) age 70–82 years, (2) total cholesterol 4.0 – 9.0 mmol/L, (3) transient ischemic accident, stroke, myocardial infarction, arterial surgery or amputation due to vascular disease >6 months prior to study entry, (4) ≥1 risk factors for vascular disease: hypertension or currently receiving drug treatment, current smoker, fastening blood glucose > 7 mmol/L or known diabetes mellitus. Subjects with poor cognitive function at baseline (Mini Mental State Examination (MMSE) < 24) were excluded. More detailed exclusion criteria have been described elsewhere [20].

Inclusion of participants for the nested MRI substudy has been described elsewhere [21]. Participants were included from the Dutch sample of the PROSPER Study. The Medical Ethics Committee of the Leiden University Medical Center approved the study. A total number of 554 study participants had a baseline MRI scan and cognitive testing at both baseline and 3.3 year (range 2.9 to 3.8 year) follow-up. Magnetization transfer imaging was introduced to the scan protocol halfway during subject inclusion and performed in the last 193 consecutive subjects at baseline. In this retained sample, data on cognitive testing at baseline and 3.3 year follow-up were available in all individuals. There was no missing data nor outliers.

### Measures of cognitive function

Cognitive function was assessed at baseline and subsequently at follow-up. MMSE is widely used to screen for cognitive dysfunction [22]. As MMSE scores were used as exclusion criteria, they are not reported in association with MRI markers. We used the abbreviated Stroop Color Word Test to assess executive functioning and attention, and measured the time to complete the color interference. All study participants were screened for color blindness.

Memory function was evaluated with the Picture Word Learning Test (PWLT), which consists of 12 pictures which subjects should recall in any order. Accordingly, we used the average number of items reported correctly in 3 conditions requiring immediate recall (immediate PWLT) and the sum of the number of items correctly recalled in the delayed condition (delayed PWLT). With the Letter Digit Coding Test (LDCT), which evaluates speed of processing of general information and tests visual scanning, visual memory, perception, visuoconstruction and motor functions, we assessed the number of correct digits. Descriptions of the cognitive tests and the procedures have been reported previously [23]. High test/re-test correlations for the STROOP and LDCT and acceptable test/re-test correlations for the immediate and delayed PWLT have been reported [23]. A small but clinically relevant improvement in cognitive test performance has been observed after testing twice with two weeks in between. By using parallel versions for the PWLT and the LDCT and redundant for the Stroop test, the possibility that study participants had remembered specific material from a test version can be ruled out. It has been suggested that practice effects should be taken into account when following the course of cognitive functions in individuals over time [23].

Depressive symptoms were measured at baseline with the 15-item Geriatric Depression Scale (GDS-15) [24]. We defined depression as a score of 4 points or more, and no depression as a score of 3 points or less on the GDS-15.

### MRI scanning

Imaging was performed on a Philips Gyroscan Intera ACS-NT 1.5T MR scanner (Philips Medical Systems, Best, The Netherlands). Axial proton-density (repetition time [TR]/echo time [TE] 2500/30 msec), T2-weighted (TR/TE 2500/120 msec), and fluid-attenuated inversion recovery (FLAIR; TR/inversion time/TE 8000 msec/2000 msec/120 msec) images were acquired with the following parameters: field of view (FOV) 220 mm, matrix 256 × 256, and 22 6-mm slices with 0.6-mm slice gap. Magnetization transfer (three-dimensional gradient-echo pulse sequence, slice thickness = 5 mm; 28 slices, no gap; TR/TE = 106/6; FA = 12^0^; FOV = 220 mm; matrix = 56 × 256; 1100 Hz below water frequency saturation pulse) images were obtained in all subjects. Raw magnetization transfer scans were split into the M0-sequence, which was acquired without the saturation pulse and the M1-sequence, which was acquired after application of a saturation pulse.

Intracranial volume, brain tissue volume, and total white matter hyperintensity volume were automatically quantified on T2-weighted and FLAIR images using Software for Neuro-Image Processing in Experimental Research [25]. We defined brain atrophy (%) as follows: [(intracranial volume – brain tissue volume)/intracranial volume] × 100 % [26].

T1-weighted images were skull-stripped using brain extraction tool [27] and subsequently segmented using FMRIB’s automated segmentation tool [28] and FMRIB’s integrated registration and segmentation tool [29], resulting in individual brain masks. The T1 weighted images were subsequently registered to the non-saturated M0 image using FMRIB’s registration tool, and the transformation matrix of this registration was saved.

In magnetization transfer imaging, MTR is the most commonly used measure, reflecting the efficiency of magnetization exchange between protons in tissue compared with surrounding water [30]. Individual MTR maps were calculated voxel by voxel following the equation MTR = (M0-M1)/M0. Low MTR is usually considered indicative of damage to myelin and other cellular structures. We evaluated both gray and white matter together, yielding a measure of overall brain damage. The frequency distribution of all MTR values was displayed as histograms. A bin size of 0.01 and 100 bins was used. To reduce partial-volume effects, a threshold of 0.20 was used to remove any remaining voxels that might still be contaminated with cerebrospinal fluid. The peak height of the histogram indicates the number of voxels that show the most common MTR value per region of interest [15]. Region of interest was defined as total brain parenchyma (gray and white matter) in our study. In healthy individuals, the histogram is characterized by a single sharp peak, indicating that normal brains are relatively homogeneous in terms of MT imaging characteristics. Decreased MTR peak height may be indicative of brain tissue decline [15]. It has been shown previously that MTR histogram peak height is a sensitive marker of changes in brain tissue integrity [31–34]. The peak height of the MTR histogram was divided by the number of voxels of brain tissue to normalize for brain size [15]. MT-MRI measures did not exceed - 3 or + 3 standard deviations.

### Statistical analysis

Data are presented as mean with standard deviation, unless stated otherwise. MMSE scores at baseline and follow-up were compared using paired-samples *T* test. To assess cross-sectional associations between tertiles of MTR peak height and cognitive test scores, general linear model multivariate analysis was performed with two-step modeling. The first model included age and education as covariates and sex as fixed factor. Estimated regression coefficients are reported for this model. Next, we used a model that adjusted for age, sex, education, systolic and diastolic blood pressure, body mass index, smoking, history of vascular disease, individual total white matter lesion load, and brain atrophy. Tertiles of MTR were obtained by a tertiary split of the numerical MTR variable, thereby dividing the data into three categories of equal number. Scores were determined at the 33^rd^ and 66^th^ percentiles.

A repeated measures general linear model was used to investigate whether tertiles of MTR peak height at baseline relate to cognitive test performance at baseline and 3.3-year follow-up. We defined baseline and follow-up cognitive test performance as within-subjects variables and tertile of baseline MTR peak height as between-subjects factors. Post hoc testing was performed with Bonferroni correction. For statistical analyses, Statistical Package for the Social Sciences (SPSS) software for windows (version 20.0) was used. *P* values less than 0.05 were considered statistically significant.

## Results

The current study cohort consisted of 193 subjects. There were 105 men and 88 women with a mean ± standard deviation age of 77.6 ± 3.2 years. Subject characteristics are shown in Table 1. MMSE scores at baseline and follow-up were 28.1 ± 1.5 and 28.4 ± 2.1, respectively, *p* = 0.025. Eight individuals declined into possible dementia according to MMSE scores at follow-up (e.g. MMSE score < 24). Median GDS-15 score at baseline was 1 (interquartile range 0–2). Six individuals (3 %) had a GDS-15 score ≥ 4 points. Cross-sectional associations between MTR peak height and cognitive function are shown in Table 2. Results from general linear model multivariate analysis showed a negative association between MTR peak height and test results on the STROOP test (unstandardized β = −3.9, *p* = 0.002). In this model, estimated regression coefficients are β = 1.3 (*p* < 0.001) for age and β = −1.6 (*p* < 0.001) for education. In the fully adjusted model, association between MTR peak height and STROOP test remained significant (β = −0.27, *p* = 0.045). There was a positive association between MTR peak height and test results on the immediate PWL test (β = 0.33, *p* = 0.004) after adjusting for age, sex, and education. In this model, estimated regression coefficients are β = −0.11 (*p* < 0.001) for age and β = 0.11 for education (*p* = 0.001). In the fully adjusted model, association between MTR peak height and immediate PWL test attenuated (β = 0.24, *p* = 0.058). There was a positive association between MTR peak height and test results on the delayed PWL test (β = 0.59, *p* < 0.001) after adjusting for age, sex, and education. In this model, estimated regression coefficients are β = −0.14 (*p* = 0.001) for age and β = 0.10 for education (*p* = 0.027). In the fully adjusted model, association between MTR peak height and delayed PWL test remained significant (β = 0.48, *p* = 0.007). There was a positive association between MTR peak height and test results on the LDC test (β = 1.6, *p* < 0.001) after adjusting for age, sex, and education. In this model, estimated regression coefficients are β = −0.36 (*p* < 0.001) for age and β = 0.85 for education (*p* < 0.001). In the fully adjusted model, association between MTR peak height and the LDC test remained significant (β = 1.1, *p* = 0.006).

**Table 1:** Baseline characteristics

Continuous variables	Values
Subjects, n	193
Age in years	77.6 (3.2)
Education, age at leaving school	15.2 (2.8)
Body mass index in kg/m^2^	26.8 (3.5)
Systolic blood pressure, mm Hg	158.4 (22.3)
Diastolic blood pressure, mm Hg	83.6 (10.0)
Fasting glucose, mmol/L	5.9 (1.7)
Total cholesterol, mmol/L	5.0 (1.1)
High density lipoprotein cholesterol, mmol/L	1.3 (0.4)
Triglycerides, mmol/L	1.4 (0.7)
Baseline mini mental state examination score, points	28.4 (2.1)
Baseline stroop test score, seconds	56.6 (23.4)
Immediate picture-word learning test score, words	10.1 (2.1)
Delayed picture-word learning test, words	10.9 (3.0)
Letter-digit coding test score, digits/min	26.0 (7.0)
MRI features	
Grey matter in mL	465.6 (59.1)
White matter in mL	609.3 (70.2)
Brain atrophy, percentage	26.1 (3.2)
White matter hyperintensity volume in mL	5.1 (9.5)
Categorical variables	
Male, n (%)	105 (54)
Current smoking, n (%)	28 (15)
History of diabetes mellitus, n (%)	41 (21)
History of transient ischemic attack or stroke, n (%)	29 (18)
Ischemic cortical infarction, n (%)	7 (4)
Lacunar infarction, n (%)	9 (5)
History of myocardial infarction, n (%)	28 (15)
Use of anticholinergics, n (%)	10 (5)
Use of antiepileptics, n (%)	0 (0)
Use of antipsychotics, n (%)	0 (0)
Use of benzodiazepines, n (%)	10 (5)

Values are means (standard deviation) unless stated otherwise

**Table 2:** Cross-sectional association between magnetization transfer ratio peak height and cognition

	Tertile of MTR peak height				
	Lower	Middle	Upper	β	*P*-value
STROOP	63.6 (29.3 – 189.3)	55.4 (29.4 – 105.3)	49.9 (30.0 – 98.3)	−0.27	0.045
PWLT immediate	9.7 (3.0 – 15.0)	9.8 (4.0 – 15.0)	10.9 (6.0 – 13.3)	0.24	0.058
PWLT delayed	10.1 (3.0 – 15.0)	10.8 (1.0 – 15.0)	12.1 (7.0 – 15.0)	0.48	0.007
LDCT	23.8 (9.0 – 40.0)	25.5 (11.0 – 36.0)	28.7 (14.0 – 43.0)	1.1	0.006

Absolute values for cognitive function tests per tertile of MTR peak height are shown (mean [minimum - maximum]). *P*-values and unstandardized β are shown for general linear model multivariate analysis, adjusting for age, sex, education, systolic and diastolic blood pressure, body mass index, smoking, history of vascular disease, individual total white matter lesion load, and brain atrophy

*Stroop* Stroop test, time in seconds to complete test, *PWLT* Picture Word Learning Test, number of items correctly recalled in immediate or delayed condition. *LDCT* Letter Digit Coding Test, number of correct digits

**Fig. 1:**
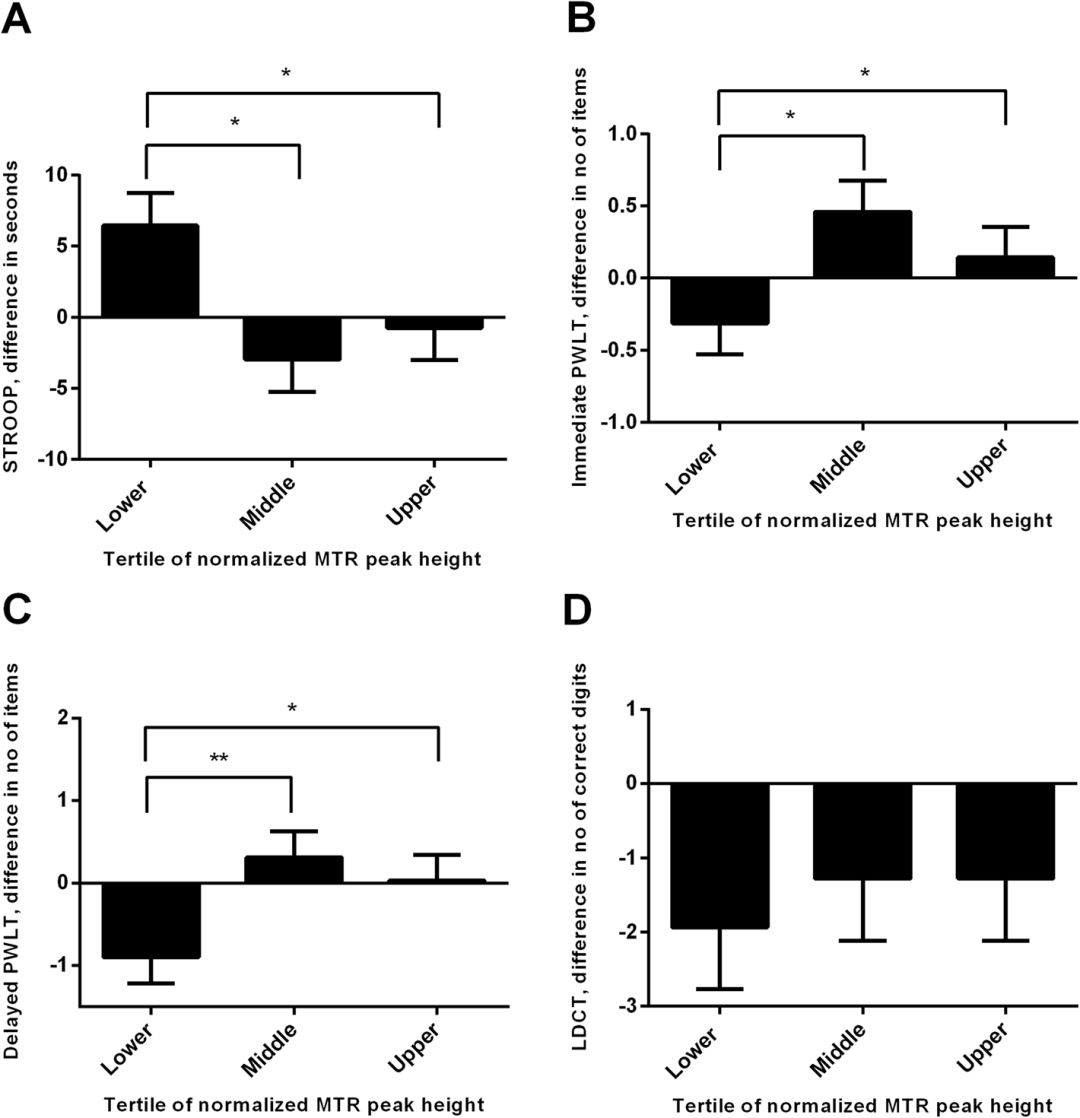
Difference in Cognition at 3.3-year Follow-Up versus Baseline across Tertiles of MTR peak height. **a**, STROOP test, difference in time (seconds) to complete test; (**b** and **c**): immediate and delayed Picture Word Learning Test (PWLT), difference in the average number of items reported correctly in 3 conditions requiring immediate recall (immediate PWLT) and the sum of the number of items correctly recalled in the delayed condition (delayed PWLT); (**d**): Letter Digit Coding test (LDCT), difference in the number of correct digits. Bar graphs represent means ± standard error of the mean. *P*-values are from repeated measures general linear model with post hoc Bonferroni test, which was used to investigate whether tertiles of MTR peak height at baseline relate to cognitive test performance at baseline and 3.3-year follow-up. * *P* < 0.05 and ** *p* < 0.01

Difference in cognitive test performance at 3.3-year follow-up versus baseline across tertiles of MTR peak height is shown in Fig. 1. General linear model repeated measures analysis showed that individuals with low MTR peak height at baseline required more time to complete the STROOP test compared to subjects with intermediate MTR peak height (mean time to complete the test at baseline and follow-up, lower versus middle tertile of MTR peak height: 61.6 versus 52.7 s, *p* = 0.019) and compared to subjects with high MTR peak height (lower versus higher tertile, 61.6 versus 53.7 s, *p* = 0.046). For individuals with low MTR peak height, average number of items reported correctly on the immediate PWLT was lower compared to individuals with intermediate MTR peak height (average number of items reported correctly at baseline and follow-up, lower versus middle tertile, 9.5 versus 10.4 items, *p* = 0.023) and compared to individuals with high MTR peak height (lower versus higher tertile, 9.5 versus 10.3 items, *p* = 0.032). Similarly, for individuals with low MTR peak height, average number of items reported correctly on the delayed PWLT was lower compared to individuals with intermediate MTR peak height (lower versus middle tertile, 10.1 versus 11.5 items, *p* = 0.004) and high MTR peak height (lower versus higher tertile, 10.1 versus 11.3 items, *p* = 0.012).

The average number of correct digits reported at baseline and follow-up on the LDCT was not different across tertiles of MTR peak height.

In none of the cognitive domains a difference in test performance at baseline and follow-up was found between subjects with intermediate and high MTR peak height.

## Discussion

The main finding of our study is that MTR peak height relates to cognitive test performance at baseline and 3.3-year follow-up in an older population at risk for vascular disease. MT-MRI nowadays has become increasingly useful in detecting microstructural cerebral changes that are correlated with cognitive decline in a variety of neurodegenerative diseases [10–17]. However, the cause and effect of the relation between diminished cognition and MTR changes in the brain in cross-sectional study designs remains unsolved. The present study aims at investigating the association between MTR and two longitudinal measures of cognition in neurologically asymptomatic older individuals at increased risk for vascular disease.

Few studies investigated the association between MTR and cognition in neurologically asymptomatic people. One recent study of an older population showed that lower MTR is related to memory impairment, executive dysfunction, and impaired motor skills [18].

In contrast, two other studies found no relation between brain MTR and cognition in healthy subjects [9, 35]. Our data unequivocally show that MTR peak height is cross-sectionally associated with cognition, as assessed by the STROOP test, the delayed Picture Word Learning test, and the Letter Digit Coding test. Although we included neurologically asymptomatic subjects in the present study, subjects were included from the PROSPER Study, which implies that all subjects had an increased vascular risk profile. It is well known that an increased vascular risk profile is the most important factor in developing cerebral damage related to small vessel disease, white mater lesions, and atrophy [36]. Both white mater lesions and atrophy directly affect MTR peak height [9]. It should be realized that extensive vascular damage such as large confluating white matter lesions, cortical ischemic or haemorrhagic infarcts strongly affect the relationships described in the present study. To reduce the confounding effects of both vascular risk factors and the presence of manifest vascular brain damage, analyses were corrected for both factors. Additionally, to reduce potential partial volume effects in calculating MTR values brain parenchyma was eroded by one MTR voxel in all three dimensions.

In addition to the associations found cross-sectionally, our data show that individuals with low MTR peak height at baseline performed worse on the STROOP test and the immediate and delayed PWLT compared to individuals with intermediate or high MTR peak height at baseline and follow-up testing. It might be the case that subjects with low MTR peak height at baseline develop a faster cognitive decline than subjects with intermediate or high MTR peak height. On the other hand, our data do not show that cognitive test performance is different in individuals with high MTR peak height compared to individuals with intermediate MTR peak height. Our data indicate a J-shape relation between MTR peak height and cognition with low MTR peak height reflecting greatest change in cognition. It can be speculated that in subjects with low MTR peak height, MTR reflects irreversible microstructural damage that is not present in the other tertiles.

MTR imaging has been used to probe the integrity of macromolecular proteins and phospholipids in the brain. Post mortem imaging and histopathology studies of multiple sclerosis have shown that in white matter lower MTR is associated with axonal loss and myelin compromise [37, 38]. In gray matter, reduction in dendritic density and neuronal size or number and damage to cell membranes may collectively or independently lead to decreased MTR [39]. We used whole brain MTR analyses, yielding a measure of overall brain tissue integrity. It should be noted that the lack of anatomical specificity limits the interpretation of the source of differences in MTR. Still, our results indicate that MTR as a marker of macromolecular degeneration relates to cognition.

The following study limitations should be considered. First, subject inclusion for this study was restricted to subjects enrolled in the PROSPER Study. Therefore, our study cohort consists of older subjects with or at increased risk for vascular disease which may limit extrapolation to the general population. However, with population aging, and given the high prevalence of vascular risk factors in the elderly, our study cohort in fact represents a substantial part of the aging population.

Second, having only two longitudinal measurements has limited our study to estimate linear change in cognition only [40] whereas more than three measurement occasions would have enabled investigation of nonlinear change.

Third, it has been proposed that accumulation of subcortical iron is a risk factor for neuronal and cognitive decline in normal aging [41]. In addition, brain iron may affect automated brain segmentation procedures and MTR measures [42, 43]. Therefore, in general, differential iron distribution in the brain may constitute bias that should be considered in computational anatomy studies of ageing and neurodegeneration.

Finally, longitudinal stability of MTR measures in our study is unknown which is a study limitation.

## Conclusions

MTR peak height relates to cognitive test performance at baseline and 3.3-year follow-up in an older population at risk for vascular disease. MTR histogram peak height may be a potentially useful marker of cognition in future clinical intervention studies.

## Additional file

Additional file 1:
**List of the individual ethics committees that gave approval for the Prospective Study of Pravastatin in the Elderly at Risk study.** (DOCX 13 kb)
