# Supplementary material for: Association between changes in brain microstructure and cognition in older subjects at increased risk for vascular disease
Source: BMC Neurol. 2015 Aug 7;15:133. doi: 10.1186/s12883-015-0396-z (PMC4545822; doi:10.1186/s12883-015-0396-z)
Supplement: Additional file 1: — List of the individual ethics committees that gave approval for the Prospective Study of Pravastatin in the Elderly at Risk study. (DOCX 13 kb) [file 12883_2015_396_MOESM1_ESM.docx]

**List of the individual ethics committees that gave approval for the Prospective Study of Pravastatin in the Elderly at Risk study.**United Kingdom

- Greater Glasgow Community/Primary Care Local Research Ethics Committee Dumfries
- Galloway Health Board Local Research Ethics Committee Argyll
- Clyde Health Board Local Research Ethics Committee Lanarkshire Research Ethics Committee

Ireland

- Research Ethics Committee of the Cork Teaching Hospitals

Netherlands
- Medical Ethical Committee of the Leiden University Medical Center
